# Validation of a Novel Double Control Quantitative Copy Number PCR Method to Quantify Off-Target Transgene Integration after CRISPR-Induced DNA Modification

**DOI:** 10.3390/mps5030043

**Published:** 2022-05-25

**Authors:** Brit-Maren Michaud Schjeide, Maren Schenke, Bettina Seeger, Gerhard Paul Püschel

**Affiliations:** 1 Department of Nutritional Biochemistry, Institute of Nutritional Science, University of Potsdam, 14558 Nuthetal, Germany; gpuesche@uni-potsdam.de; 2 Department of Nutritional Biochemistry, Faculty of Life Sciences: Food, Nutrition and Health, University of Bayreuth, 95326 Kulmbach, Germany; 3 Research Group Food Toxicology and Alternative/Complementary Methods to Animal Experiments, Institute for Food Quality and Safety, University of Veterinary Medicine Hannover, 30173 Hannover, Germany; maren.schenke@tiho-hannover.de (M.S.); bettina.seeger@tiho-hannover.de (B.S.)

**Keywords:** CRISPR editing validation, copy number analyses, homology-directed repair, homologous recombination deficiency

## Abstract

In order to improve a recently established cell-based assay to assess the potency of botulinum neurotoxin, neuroblastoma-derived SiMa cells and induced pluripotent stem-cells (iPSC) were modified to incorporate the coding sequence of a reporter luciferase into a genetic safe harbor utilizing CRISPR/Cas9. A novel method, the double-control quantitative copy number PCR (dc-qcnPCR), was developed to detect off-target integrations of donor DNA. The donor DNA insertion success rate and targeted insertion success rate were analyzed in clones of each cell type. The dc-qcnPCR reliably quantified the copy number in both cell lines. The probability of incorrect donor DNA integration was significantly increased in SiMa cells in comparison to the iPSCs. This can possibly be explained by the lower bundled relative gene expression of a number of double-strand repair genes (*BRCA1*, *DNA2*, *EXO1*, *MCPH1*, *MRE11*, and *RAD51*) in SiMa clones than in iPSC clones. The dc-qcnPCR offers an efficient and cost-effective method to detect off-target CRISPR/Cas9-induced donor DNA integrations.

## 1. Introduction

Botulinum neurotoxin (BoNT) is widely used as a therapeutic [1]. The activity of the toxin is frequently assessed by the ethically disputable mouse lethality assay [2]. Cell-based alternative methods have been established but must be improved to enhance sensitivity [3]. To this end, the coding sequence of a reporter luciferase (*Gaussia* luciferase, GLuc) used in a recently developed neuroblastoma cell-based functional assay [4,5,6] was incorporated into a genetic safe harbor of induced pluripotent stem cells (iPSCs), which can be differentiated into highly sensitive motor neurons [7] by the CRISPR/Cas9 method.

The CRISPR/Cas9 method allows the incorporation of foreign DNA at a targeted genomic locus by introducing a double-strand break (DSB) at a specific DNA site using a single-guide RNA-directed endonuclease. The double-strand break is then repaired by the homology-directed recombination (HDR) repair system. Taking advantage of HDR allows the incorporation of foreign DNA flanked by homology arms at the site of the double-strand break [8,9,10].

**Figure 1:**
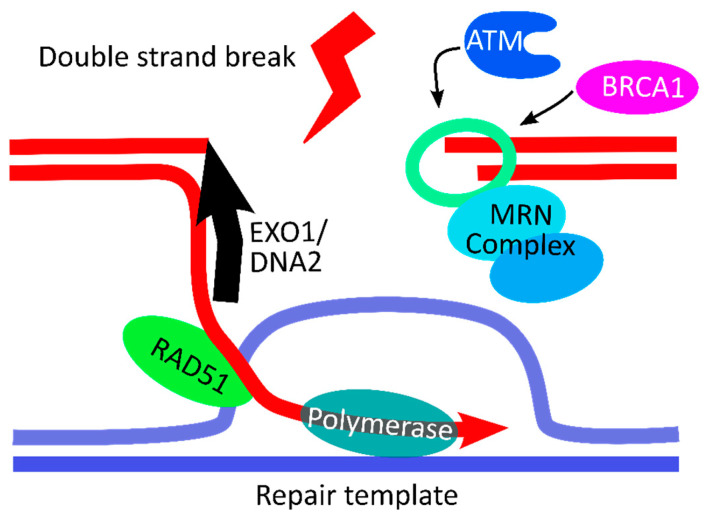
Illustration of key steps of the homology-directed recombination mechanism. Homology-directed repair is initiated as ATM binds to the double-strand break (DSB) and attracts BRCA1 to the DSB, along with the MRN (MRE11, RAD50, NBS1) Complex. The protein complex, along with EXO1 and DNA2, generates 3′-single-stranded-DNA ends on each side of the DSB. The recombinase RAD51 works in concert with other proteins to search for homologous DNA, which is then invaded by the free 3′ single-stranded DNA ends. Once found, the repair template is unwound, and a DNA polymerase synthesizes a new DNA strand. Finally, double-strand-break repair uses the 3′-overhangs to create a double Holliday junction linking the two homologous regions, normally resulting in a crossover product [11,12,13,14]. Figure created using Inkscape (https://inkscape.org (accessed on 19 May 2022), Brooklyn, NY, USA).

DSB repair is a multilevel coordinated response to DNA damage resulting in the transcriptional activation of a large set of genes [11,12,13,14]. Examples of key players in HDR are ataxia-telangiectasia-mutated gene (ATM), breast cancer 1 gene (BRCA1), the MRN Complex (consisting of double-strand break repair nuclease (MRE11), double-strand break repair protein (RAD50), and nibrin (NBS1)), as well as exonuclease 1 (EXO1), DNA replication helicase/nuclease 2 (DNA2), and RAD51 recombinase (RAD51) (Figure 1). Alterations to any of the genes involved in HDR can lead to homologous recombination deficiency (HRD), a condition in which DSB repair is diminished and which is a hallmark of cancer cells and associated with multiple cancer types [14,15]. As opposed to tumor-derived cells, iPSCs have been shown to be hypersensitive to DNA damage, responding by either entering apoptosis or activating checkpoint signaling and initiating elevated expression of genes associated with DSB repair [16].

Despite the highly efficient double-strand repair system, a major limitation of the CRISPR/Cas9 genetic editing method is that the insertion of the foreign DNA may also occur at random sites due to spontaneous double-strand breaks or off-target cleavage by Cas9. Any established cell line must therefore be scrutinized for potential off-target integrations. The currently available techniques are, however, expensive, error prone, and of low sensitivity. Therefore, a double-control quantitative copy number PCR (dc-qcnPCR) was established to analyze the specificity of the reporter-sequence integration in the AAVS1 safe harbor locus and was tested in transgenic clones of the iPSC line IMR90-4 and neuroblastoma-derived SiMa cells in comparison.

## 2. Materials and Methods

### 2.1. Standard Cell Culture Maintenance of SiMa and IMR90-4 Cell Lines

SiMa cells [17] were cultivated in RPMI1640 GLutaMAX™ medium (Gibco, Grand Island, NY, USA) with 1% penicillin/streptomycin (P/S, PAN Biotech, Aidenbach, Germany), and 10% Fetal Bovine Serum (FBS, PAN Biotech, Aidenbach, Germany) on untreated cell culture plates. Medium was changed every 2–3 days and upon reaching 80–90% confluency, cells were split 1:15 after trypsinization (Trypsin-EDTA, Gibco, Grand Island, NY, USA). Undifferentiated IMR90-4 cells [18] were cultured on Matrigel-coated (Corning, NY, USA) plates in StemMACs™ iPS-Brew XF human cell culture medium (Miltenyi, Bergisch Gladbach, Germany) containing 1% P/S in feeder-free conditions. Medium was changed every 2 days and cells were split 1:40 upon reaching 80–90% confluency with 0.5 mM EDTA (Sigma-Aldrich, Darmstadt, Germany) in PBS. Newly split cells were supplemented with 1 µM Y-27632 ROCK inhibitor (Bertin Pharma, Montigny le Bretonneux, France). Matrigel was applied to the appropriate cell-culture plates (96/24/6-well plates, Sarstedt, Nümbrecht, Germany) and incubated at room temperature for at least 2 h before use. The Matrigel solution was prepared on ice by diluting previously prepared aliquots 1:100 with KnockOut Medium (Thermo Fisher Scientific, Waltham, MA, USA).

### 2.2. Stable Transfection of SiMa and IMR90-4 Cell Lines

The stable transfection of SiMa cells was carried out according to the TurboFect (Thermo Fisher Scientific, Waltham, MA, USA) transfection protocol with 10 µg total plasmid DNA in one well of a 6-well plate. The cells were seeded at 50,000 cells per well and transfected the following day with TurboFect transfection medium. The transfection medium was added to the adherent cells and incubated for 48 h at 37 °C. To isolate the cells harboring stably integrated donor DNA, the medium was changed to the standard RPMI1640 medium plus 1 µg/mL puromycin antibiotic (Thermo Fisher Scientific Waltham, MA, USA). The donor DNA contains the sequence for the puromycin resistance gene and those cells expressing this gene are likely also to contain the Gluc fusion sequence of interest. The RPMI1640 medium including puromycin was changed every two days. Medium change continued until clusters of healthy cells began to grow after approximately 2–4 weeks. These healthy cell clusters were trypsinized and transferred to wells of a 96-well plate as a single-cell dilution for monoclonal isolation.

The IMR90-4 transfection was carried out according to the Lipofectamine 3000 (Thermo Fisher Scientific, Waltham, MA, USA) transfection protocol. Cells were seeded in wells of a 6-well plate and the transfection began once the cells reached 60–70% confluency. The cells and transfection reagents were incubated at 37 °C for 48 h. The cells were selected for the stable genomic integration of donor DNA by removing the transfection medium and applying standard iPS-Brew medium containing 500 ng/mL puromycin. The selection medium was changed every two days. Once clonal clusters began to grow, they were split into 96-well plates for single-cell dilution and monoclonal isolation. Cells on the 96-well plate were incubated in CloneR medium at the concentrations specified by the manufacturer. The CloneR reagent (Stemcell Technologies, Cologne, Germany) was diluted in the standard iPS-Brew medium plus 500 ng/mL puromycin. CloneR supports the survival of single cells and was observed to inhibit spontaneous differentiation of the isolated clones.

### 2.3. Double-Control Quantitative Copy Number PCR

The assessment of the insert copy number in each clone was carried out by a newly developed method based upon the qPCR, as described below. Four plasmids were prepared by cloning the sequence encoding *Gaussia* luciferase (GLuc), a 200 bp fragment of the autosomal gene DNA damage-inducible transcript 3 (*CHOP*), and one each of the chromosome X (ChrX) genes hypoxanthine phosphoribosyltransferase 1 (*HPRT1*), retinoblastoma binding protein 7 chromatin remodeling factor (*RBBP7*), thymosin beta 15B (*TMSB15B*), or GATA binding protein 1 (*GATA1*) into a donor plasmid. Each plasmid is designated by the ChrX gene it contains. After stable transfection and monoclonal selection of the SiMa or IMR90-4 cell lines, genomic DNA was isolated from each clone and the GLuc sequence, as well as *CHOP* and the ChrX sequences, were amplified by qPCR with the incorporation of SYBR green (see Supplementary Table S1 for primer sequences). Each of the cloned sequences on each plasmid was amplified in parallel to the gDNA. The copy number of the GLuc sequence was normalized to the autosomal and ChrX copy numbers and the plasmid amplification rate (correcting for primer efficiency), as described below.

An example calculation using RBBP7 plasmid for insert copy number is given below. The optimization including the remaining plasmids was calculated in the same manner. The qPCR plate prepared according to this method always contained the control plasmid, the samples under investigation, and non-template controls for each sequence under investigation: GLuc, CHOP, and RBBP7. Furthermore, control HapMap gDNA samples derived from a female and a male were included in each plate and analyzed with CHOP and RBBP7 primer sets. These samples were not included in the GLuc measurement because luciferase is not encoded in the human genome.

Once cycle threshold (Ct) values for each sample and sequence were collected, the sample amplification was normalized to the plasmid amplification within each gene analyzed:

(1) $$\mathrm{normalization}\;\mathrm{within}\;\mathrm{gene}=\frac{2^{{\mathrm{Ct}}_{\mathrm{plasmid}}-{\mathrm{Ct}}_{\mathrm{sample}}}}{\mathrm{average}\;(2^{{\mathrm{Ct}}_{\mathrm{plasmid}}-{\mathrm{Ct}}_{\mathrm{plasmid}}})}$$

(2) $$\mathrm{normalization}\;\mathrm{relative}\;\mathrm{to}\;\mathrm{CHOP}=\frac{{\left(1\right)}_{\mathrm{sample}\left(\mathrm{GLuc}\;\mathrm{or}\;\mathrm{RBBP}7\right)}}{{\left(1\right)}_{\mathrm{sample}\;\left(\mathrm{CHOP}\right)}}$$

For these calculations, the primer efficiency was normalized by comparing each gene of interest with the same plasmid. Furthermore, the normalization relative to CHOP designated the exact Ct value, within each sample, which is equals to two since autosomal genes possess two alleles. The secondary normalization to RBBP7 verified that the CHOP normalization results in the ChrX gene equals two in females and one in males. Female and male gDNA were always included as a positive control during qPCR analyses.

### 2.4. Insert Confirmation (PCR)

The insert confirmation was carried out using a pair of primers designed to flank the AAVS1 safe-harbor locus, at which the Cas9 endonuclease induces the double-strand break in the gDNA (see Supplementary Table S1 for primer sequences). The PCR products were amplified using Phusion™ High-Fidelity DNA Polymerase (Thermo Fisher Scientific, Waltham, MA, USA) and separated on an agarose gel and the amplicons were excised, gel purified, and Sanger sequenced. The sequencing step confirms the amplification of the correct product, and excludes any mutations that might have been incorporated into the gDNA during the homology-driven recombination.

### 2.5. Gene-Expression Analysis

For gene-expression analysis, RNA extraction was carried out with the PeqGOLD Total RNA Kit (VWR, Peqlab, Darmstadt, Germany) according to the manufacturer’s instructions. A total of 2 µg RNA was reverse transcribed to cDNA using Reverse Transcriptase (Thermo Fisher Scientific, Waltham, MA, USA) with dNTPs (Thermo Fisher Scientific, Waltham, MA, USA) and Oligo (dt) 18 primers (Thermo Fisher Scientific, Waltham, MA, USA). Twenty nanograms of cDNA were amplified per qPCR reaction with Maxima SYBR green qPCR master mix (Thermo Fisher Scientific, Waltham, MA, USA). The HRD-associated genes *BRCA1*, *DNA2*, *EXO1*, *MCPH1*, *MRE11*, and *RAD51* were analyzed along with the reference genes *PPIA* and *RPS23*. Samples were run in triplicate and relative gene expression was calculated with the 2^−ΔΔCt^ (delta delta cycle threshold) method using the geometric mean of expression of the reference genes peptidylprolyl isomerase A (*PPIA*) and ribosomal protein S23 (*RPS23*) [19]. The formula to calculate the relative fold gene expression against the reference sample is as follows, whereby goi = gene of interest, and ref gene = reference gene:

$$\mathrm{relative}\;\mathrm{fold}\;\mathrm{gene}\;\mathrm{expression}=\frac{2^{{\mathrm{Ct}\;\mathrm{goi}}_{\mathrm{control}}-{\mathrm{Ct}\;\mathrm{goi}}_{\mathrm{sample}}}}{2^{{\mathrm{Ct}\;\mathrm{ref}\;\mathrm{gene}}_{\mathrm{control}}-{\mathrm{Ct}\;\mathrm{ref}\;\mathrm{gene}}_{\mathrm{sample}}}}$$

### 2.6. Statistical Analyses

Statistical differences in gene expression were analyzed using the unpaired two-tailed *t*-test at 95% confidence level in GraphPad Prism (GraphPad Software, San Diego, CA, USA). Distributions of the targeted insertion rate and homozygosity rate were evaluated with two-sided chi-square analysis at 95% confidence level in GraphPad Prism. Functional correlation between ranked relative gene expression and transgene insert copy number was analyzed by the Pearson correlation coefficient, 95% confidence interval, two-tailed test in GraphPad Prism.

## 3. Results

### 3.1. Insert Confirmation

**Figure 2:**
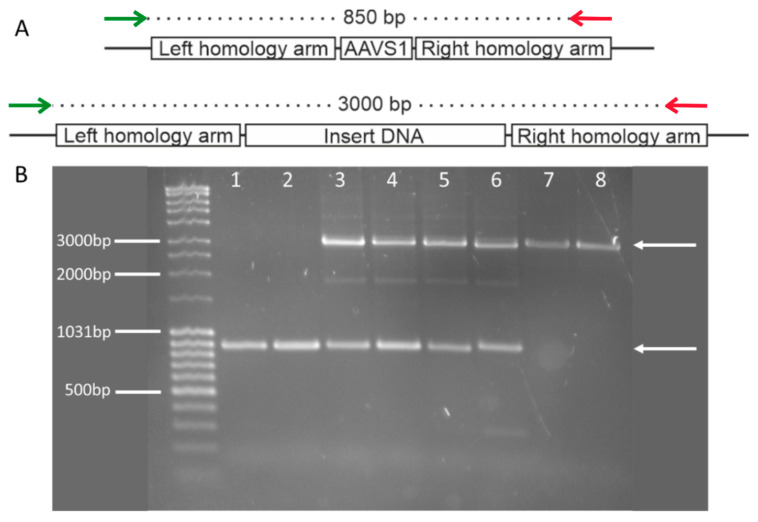
Summary of insert confirmation PCR (**A**) Summary of the Cas9 induced double-strand break, the consequential integration of the donor DNA into the AAVS1 safe harbor locus by homology-driven recombination, and the locations of the primer pair designed to amplify the genetic area surrounding the AAVS1 safe-harbor locus. The forward primer (green) is located in the flanking region 5′ of the left homology arm, the reverse primer (red) is located within the right homology arm. The wild-type (WT) confirmation amplicon has an expected product size of 850 bp and the insert confirmation amplicon has an expected product size of 3000 bp. (**B**) Amplification of the region surrounding the AAVS1 safe harbor locus in non-transfected SiMa (lane 1), non-transfected IMR90-4 (lane 2), SiMa clones 1–3 (lanes 3–5), and IMR90-4 clones 1–3 (lanes 6–8). The appropriate amplicon sizes are marked by white arrows and are found at 850 bp for the WT allele and 3000 bp for the allele containing the insert.

The successful insertion of the luciferase into the AAVS1 safe harbor locus was verified by PCR using a forward primer located in the 5′-flanking region of the left homology arm and a reverse primer located at the 3′ end of the right homology arm. The PCR would yield an 850 bp product if no insertion occurred, whereas a 3000 bp product was generated only after insertion into the safe harbor (Figure 2A). In non-transfected cells only the 850 bp band was visible. In transfected cells, either both 850 bp and 3000 bp bands were visible if a heterozygous recombination occurred. If the recombination occurred in both alleles, a single band at 3000 bp was observed (Figure 2B).

**Figure 3:**
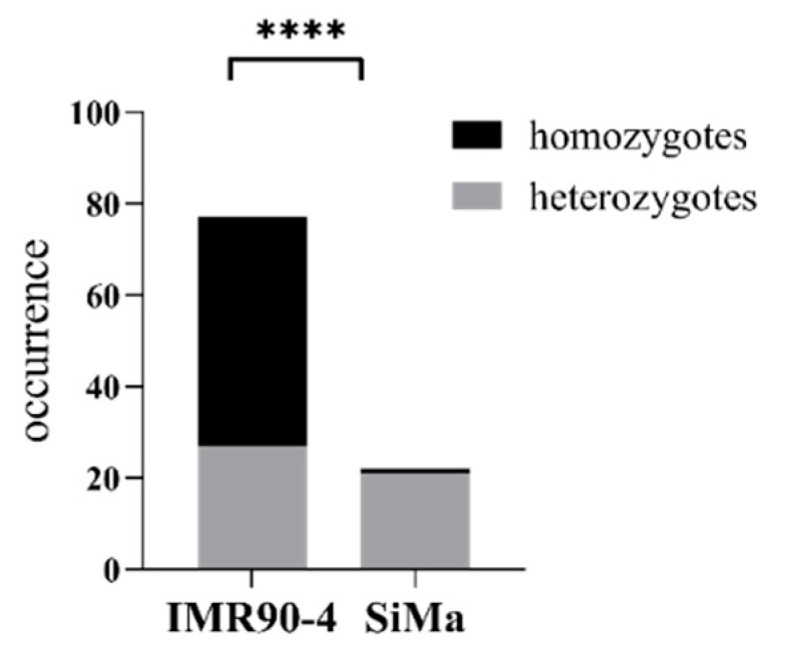
Donor DNA insertion success rate. Absolute occurrence of homozygous integration events vs. heterozygous integration events at the AAVS1 safe harbor locus. Frequency analysis measured by Χ^2^, **** = *p* > 0.0001.

Seventy-seven IMR90-4 clones were identified that had integrated the luciferase into the safe harbor. Of these, 50 clones (65%) contained the donor DNA insert in both alleles and could be assessed as homozygotes, whereas the remaining 27 clones only integrated the donor DNA into one allele. In contrast, only one clone of 22 SiMa clones (<5%), contained an insertion of the luciferase donor DNA in both alleles of the safe harbor. The rate of homozygous insertions vs. heterozygous insertions was significantly higher in IMR90-4 clones than in SiMa clones (Figure 3).

### 3.2. Establishment of Technique for Copy-Number Quantification Using qPCR Analysis

A frequently used technique to exclude off-target insertion of a transgene is the Southern blot [20]. However, several SiMa clones into which the GLuc sequence was incorporated randomly into the genome by a standard transfection protocol failed to give an unequivocal signal in Southern blots despite the functional proof of stable luciferase expression (Supplementary Figure S1). Since the Southern blot apparently was prone to false-negative results, a more sensitive and reliable method was needed. Therefore, a quantitative PCR to determine the copy number of a specific sequence was established. This PCR was calibrated with endogenous genes that are coded by autosomes (*n* = 2) and X-Chromosomes (ChrX, *n* = 1 in males, *n* = 2 in females).

**Figure 4:**
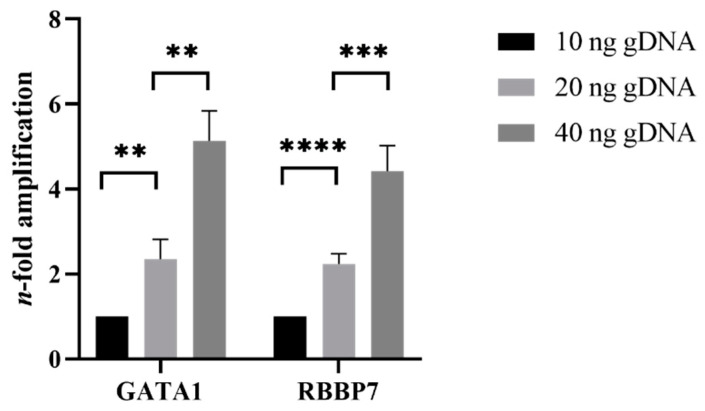
Proof-of-concept gDNA doubling and quadrupling in male samples. Amplification of 10 ng, 20 ng, and 40 ng male gDNA, doubling calculation using the ΔCt method. Values displayed mean ± SD, statistical differences are determined with the two-tailed, unpaired *t*-test. *n* = 3, ** = *p* < 0.01, *** = *p* < 0.001, **** = *p* < 0.0001.

First, it was confirmed that the qPCR method faithfully detected single-copy changes in small amounts of gDNA. Two different genes, *GATA1* and *RBBP7*, were amplified from 10 ng, 20 ng, and 40 ng gDNA. The fold difference between sample amplification was calculated using the ΔCt method, whereby the smallest amount of gDNA was used as the reference for each respective gene. The comparison of double the gDNA resulted in double the copy number; therefore, the qPCR method appeared to be sensitive enough to detect the difference between one and two copies (Figure 4).

**Figure 5:**
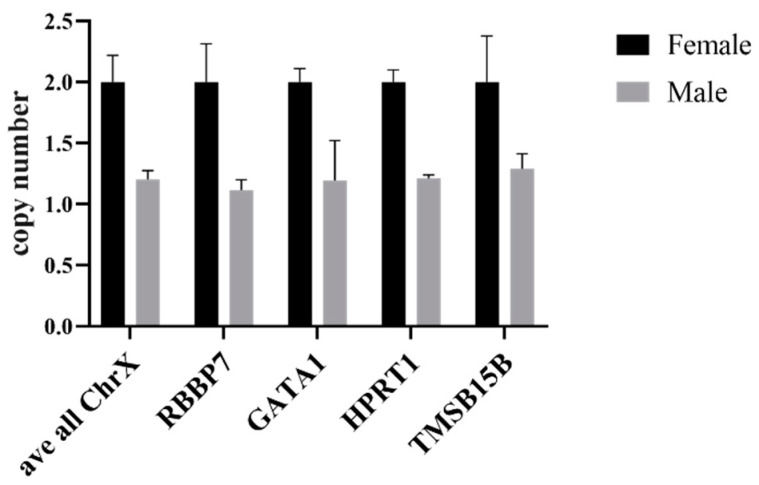
qPCR analysis of ChrX genes in female and male samples. Change in copy number calculated using the ΔCt method in ChrX normalized to autosomal gene CHOP. Values displayed as mean ± SD, *n* = 3.

The efficacy of quantitative PCR was further confirmed by comparing the copy numbers of X-chromosomal genes of male and female gDNA to autosomal genes. The copy numbers of four ChrX genes (*RBBP7*, *GATA1*, *HPRT1*, *TMSB15B*) were compared to the copy number of one autosomal gene, *CHOP*. The copy numbers of all four of the ChrX genes were twice as high in female samples compared to the male samples (Figure 5). This test indicates that the qPCR method should be sensitive enough to detect a single change in the copy number of a gene in gDNA samples.

The normalization of the copy number and concentration are important factors to consider in the attempt to quantify gene copy numbers. However, when using and comparing multiple primer pairs to amplify various genetic segments, it is also important to consider any possible differences in primer efficiencies. For this reason, one more control step was added to this quantification technique, which can be used to normalize all three of these disrupting factors (copy number, concentrations, primer efficiencies) in the qPCR. Specifically, a plasmid was constructed to contain the autosomal gene sequence for *CHOP*, the genetic sequence for one of the ChrX genes (either *RBBP7*, *GATA1*, *HPRT1*, or *TMSB15B*), and the sequence for GLuc. This plasmid can be included and amplified with each primer pair, alongside any clone gDNA, for normalization and control purposes. The procedure to calculate the copy number based on the amplification rate of each gene from the plasmid and gDNA is described in Section 2.

### 3.3. Analysis of Donor DNA Copy Number by dc-qcnPCR

**Figure 6:**
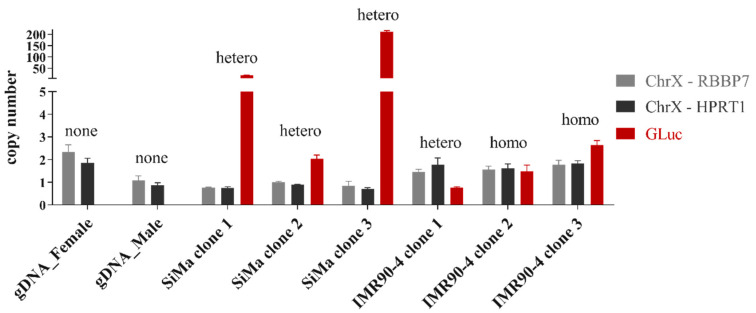
Copy numbers of RBBP7, HPRT1, and GLuc in selected clones determined by double-control quantitative copy-number PCR. Copy number calculated using the ΔCt method, whereby GLuc/ChrX is normalized to the corresponding plasmid amplification and CHOP amplification. Values as displayed mean ± SD, *n* = 3. Results of GLuc insert confirmation from Figure 2B found above each copy-number column: none—no insert detected; hetero—heterozygous clone (insert detected in one allele); homo—homozygous clone (insert detected in both alleles).

The ChrX and GLuc copy-number values were analyzed and compared according to the dc-qcnPCR method. Male and female gDNA served as controls. The copy number for both ChrX genes (*RBBP7* and *HPRT1*) in the control female was approximately two, while the copy number for both genes in the control male was approximately one. For each of the clones, the two ChrX gene copy numbers depicted in grayscale can be compared to the GLuc copy numbers colored in red. SiMa cells are derived from a male donor and only have one copy of ChrX, while the IMR90-4 cells are derived from a female donor and have two copies of ChrX. The amplification of the ChrX genes was consistent both between samples and between genes. SiMa clones 1 and 3 both had GLuc copy numbers greater than 20, indicating many off-target integration events. SiMa clone 2 had a donor DNA integration number of two, which, in conjugation with the insert confirmation PCR, was proven to include one on-target integration and one off-target integration. On the other hand, IMR90-4 clone 1 included one copy of the GLuc sequence, which corresponded to a heterozygous insertion in the AAVS1 safe harbor locus. IMR90-4 clone 2 included two copies of the GLuc sequence, corresponding to homozygous insertions into both alleles of the AAVS1 safe harbor locus. On the other hand, IMR90-4 clone 3 had a GLuc copy number greater than two, indicating at least one off-target donor DNA integration (Figure 6).

### 3.4. Defective Homology-Driven Double Strand Repair in SiMa Cells

**Figure 7:**
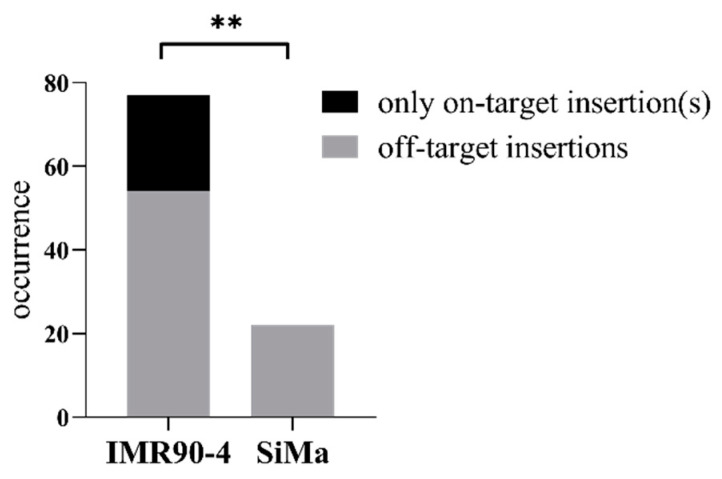
Targeted insertion success rate. Absolute occurrence of only on-target donor DNA insertions into the AAVS1 safe-harbor locus vs. occurrence of off-target donor DNA insertions in IMR90-4 and SiMa clones. Frequency analysis measured by Χ^2^, ** = *p* > 0.01.

The results of the insert confirmation and the dc-qcnPCR were combined to determine which clones contained only on-target insertions, either as hetero or homozygotes, and which clones were affected by any number of off-target insertions. Twenty-three of the IMR90-4 clones isolated were found to have insertions only at the AAVS1 safe-harbor locus with no off-target integrations, and 54 of the clones were found to have both on-target and off-target donor DNA insertions. On the other hand, not even one of the SiMa clones could be found to have only on-target insertions into the AAVS1 safe harbor. All 22 of the SiMa clones were shown to have at least one off-target integration in their genomes. The rate of on-target insertions vs. off-target insertions for the two cell-types was analyzed by chi-square and the targeted insertion success rate was found to be significantly higher in IMR90-4 clones than in SiMa clones (Figure 7).

**Figure 8:**
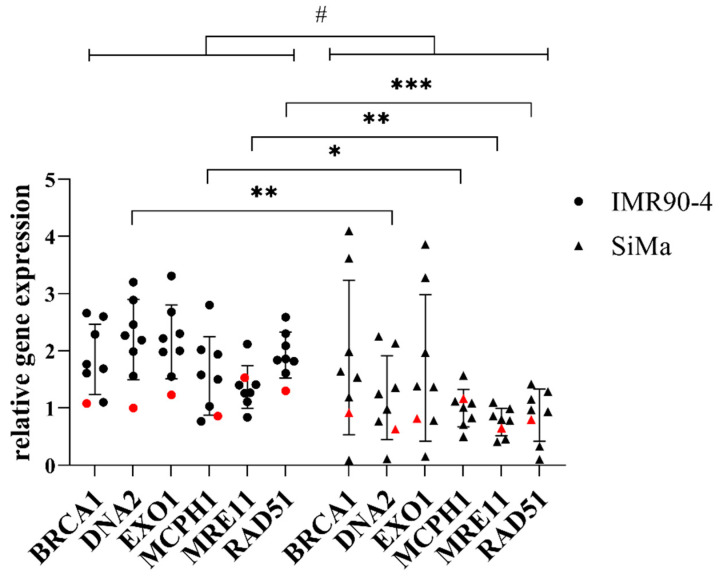
Relative gene expression of homology repair-deficiency-associated genes in IMR90-4 and SiMa clones. Expression calculated relative to non-transfected IMR90-4 and SiMa cells and reference genes *PPIA* and *RPS23*. Values shown in red correspond to non-transfected IMR90-4 and SiMa clones. Values displayed as individual clones ± SD, *n* = 7. Statistical analysis: Unpaired *t*-test IMR90-4 vs. SiMa within individual genes: * = *p* < 0.05, ** = *p* < 0.01, *** = *p* < 0.001. Unpaired *t*-test IMR90-4 vs. SiMa all genes bundled: # = *p* < 0.05.

The expression of the genes *BRCA1*, *DNA2*, *EXO1*, *MCPH1*, *MRE11*, and *RAD51* was analyzed in the IMR90-4 and SiMa clones (depicted in black, Figure 8), relative to the corresponding gene expression in non-transfected IMR90-4 and SiMa cells (depicted in red, Figure 8). There was a significantly lower expression of *DNA2*, *MCPH1*, *MRE11*, and *RAD51* in SiMa clones compared to IMR90-4 clones. There was no difference between the clones in their expression of *BRCA1* or *EXO1*. The relative gene expression of all HRD-associated genes together was significantly lower in SiMa clones than in IMR90-4 clones (Figure 8). When the clones were ranked for the expression level of the repair enzymes, a significant negative correlation between the rank and the log of the transgene copy number was observed (Supplementary Figure S2), indicating a functional correlation between double-strand-break repair efficiency and off-target transgene insertion.

## 4. Discussion

### 4.1. Analysis of Donor DNA Copy Number by dc-qcnPCR

CRISPR/Cas9 technology is a frequently used, highly efficient knock-in genetic editing tool. It takes advantage of the homology-driven DNA double-strand repair system to insert foreign DNA into a DNA double-strand break that is placed at a specific site by the high sequence specificity of the CRISPR/Cas9 endonuclease. Despite the high specificity of the endonuclease, off-target insertions may still occur and might impair cellular functions by disrupting functionally relevant genes. Therefore, cost-efficient methods to analyze possible off-target integration events during the knock-in process are needed. While the standard Southern blot [20] is a relevant method, it lacks sensitivity to reliably label a single copy of DNA of interest, especially using non-isotope-based strategies. In the current study, it failed to detect functionally proven stable integrations of Luciferase-coding DNA in SiMa cells (Supplementary Figure S1). Given the number of clones necessary to isolate and characterize for a given experiment, homology-independent genome-wide assays (such as GUIDE-seq [21], DISCOVER-seq [22], or CIRCLE-seq [23]) in combination with next-generation sequencing [24], can be cost prohibitive and an undue analytical burden for many smaller laboratories.

Significant advances have also been made in the realm of quantitative PCR, especially with the development of the digital droplet PCR, which has been shown to accurately and efficiently measure copy-number variation [25]. Unfortunately, this method requires specific instruments for droplet generation and reading, which can also be a financial strain. The double-control quantitative copy number PCR (dc-qcnPCR) method requires only standard equipment found in almost all molecular biology laboratories. Plasmids must be generated containing the DNA sequences of interest using standard bacteria transformation methods, and the resulting CRISPR-modified clones must be analyzed with qPCR and PCR. The most comparable and reasonably priced method to analyze copy-number variation is the TaqMan™ Copy Number Assay, which utilizes TaqMan™ probes in a duplex reaction to normalize the gene of interest to a reference gene [26]. This method is simple and powerful, but establishes a dependency on the manufacturer to produce the number of reactions needed and requires a new assay for each gene of interest. The dc-qcnPCR retains the flexibility to prepare plasmids with multiple sequences of interest in a quantity that will provide for thousands of reactions. This study has analyzed almost 100 CRISPR-modified clones derived from the iPSC line IMR90-4 and the neuroblastoma cell line SiMa for on- and off-target CRISPR-mediated integration events using insertion-specific PCR in combination with this newly established qPCR-based copy-number assessment tool.

qPCR can detect small variations in the amount of cDNA from which fragments are amplified [27]. Here, we show that the same instrument can be used to detect small variations in gDNA copy number. Therefore, the autosomal/dc-qcnPCR method was devised and optimized to reliably detect single copies of the transgene in clones with heterozygous insertion, as well as further increments of one of the inserted target sequence. To exclude false-negative results, it is critical to this test that the cell population is monoclonal. While any result indicating more than two copies clearly suggests the presence of off-target integrations, any result indicating two copies or less could either suggest the heterozygous or homozygous insertions or be the consequence of a contamination of transgene-containing cells with non-transfected cells. The current study excludes the possibility of contamination through the immunocytochemical detection of luciferase in all cells. With this limitation, this technique has proven to be an efficient and simple method to identify clones with only one or two copies of the donor DNA inserted into the genome. If two copies are amplified, it is possible to determine if the clone is homozygous for the insertion or if it contains one on-target insertion and one off-target insertion in conjunction with the insert confirmation PCR result. This method provides a quick, easy, and cheap in-house method to analyze off-target CRISPR-mediated integrations.

### 4.2. Defective Homology-Driven Double Strand Repair in SiMa Cells

While on-target insertion depending on CRISPR/Cas9 and homology-driven double strand repair apparently was less efficient in SiMa cells than in IMR90-4 iPSCs, i.e., only 5% versus 65% homozygous clones, respectively, off-target insertion was more likely in SiMa cells. Whereas all SiMa clones contained off-target integrations, 30% of all IMR90-4 clones were devoid of off-target insertions. Immortal tumor-derived cell lines, such as the SiMa neuroblastoma cell line, are inherently flawed in that they tend to suffer from dysregulated DNA repair mechanisms, such as homology-driven recombination, potentially favoring off-target integrations and chromosomal abnormalities [28,29,30,31,32,33,34,35]. On the other hand, off-target effects appear to be less frequent in healthy human iPSC clones with functional repair pathways [36,37].

Double-strand breaks may not only be provoked by the Cas9 nuclease but can also occur spontaneously [38]. HDR is mainly active during the S/G2 (synthesis/gap 2) phases [39]. Efficient DSB repair requires the detection of the damage involving *MRE11* [40,41]; the suppression of further breakage, and cell-cycle arrest to allow the subsequent repair steps, i.e., trimming the DNA ends, including, but not limited to *DNA2*, *EXO1*, and *BRCA1* [42,43]; and the search for and invasion of the appropriate homology sequence requiring, among others, *RAD51* [44] and *MCPH1* [45]. This results in the final steps of DNA synthesis and ligation [11,14,46]. These exemplary genes have been shown in knockdown cell-lines to be associated with decreased HDR efficiency [12], also referred to as homologous recombination deficiency.

The expression of four out of the six aforementioned genes associated with HRD was significantly lower in SiMa-derived clones than in IMR90-4-derived clones (Figure 8), while the all-around expression of all analyzed genes together remained significantly lower. Due to the downregulation of these key genes, any DSB that may occur during the transfection process may be more likely to be erroneously repaired. Therefore, the reduced levels of HRD-associated gene expression in SiMa-derived, CRISPR-modified clones support the hypothesis that the less efficient integration of the donor DNA into both alleles at the AAVS1 safe-harbor locus in the SiMa clones could be due to dysregulated DSB repair mechanisms.

On the other hand, it has been shown that gene expression related to repair mechanisms in iPSCs is generally greater than in their differentiated counterparts. Furthermore, the iPSCs were demonstrated as more likely to undergo apoptosis after suffering damage [47,48]. This could mean that the IMR90-4 cells in which multiple off-target DSBs were instigated may have undergone apoptosis, before even having the chance to incorrectly integrate the donor DNA segment. The remaining IMR90-4 cells were able to take advantage of their active HDR machinery to produce a greater number of precise integrations at the AAVS1 safe harbor. The negative correlation between the rank of cumulative HRD-associated gene expression level and the number of off-target transgene insertions (Supplementary Figure S2) supports the hypothesis that a causal relationship between intact HDR and efficient CRISPR/Cas9-driven targeted transgene insertion might exist.

Despite the intact repair pathways present in the IMR90-4 cell line, there were still some off-target events identified, suggesting that the direction of the Cas9 endonuclease to the AAVS1 safe-harbor locus was not always perfect, but still more precise in the iPSCs than in the SiMa cells. Further steps must be taken to improve the rate of on-target genetic modifications using CRISPR/Cas9. Much focus has also been placed on the Cas9 endonuclease, including genetic manipulations to improve specificity. One such example is the use of Cas9 nickases, which can be designed to make only a single-strand break in the genomic DNA. Using a pair of nickases, the enzymes can adjacently cut the gDNA locus of interest and increase the likelihood of a targeted multistep double-strand break [49,50].

## 5. Conclusions

The newly developed dc-qcnPCR offers an efficient and cost-effective method to detect copy-number variations, including the quantification of on- and off-target CRISPR/Cas9-induced donor DNA integrations. In this case, the method was successfully utilized to demonstrate the higher insertion success rate and targeted insertion success rate in iPSCs in comparison to the tumor-derived SiMa cells. The more precise CRISPR-mediated genetic editing appears to be at least partly due to differences in the expression of genes associated with homology-directed repair.

## Data Availability

Not applicable.

## Supplementary Materials

The following supporting information can be downloaded at: https://www.mdpi.com/article/10.3390/mps5030043/s1, Figure S1: Southern blot with GLuc probe at 48 °C; Figure S2: Correlation between expression level rank and log of transgene copy number, Table S1: Primers used for insert amplification, qPCR, dc-qcnPCR, and Southern blot.
