# Supplementary material for: Validation of a Novel Double Control Quantitative Copy Number PCR Method to Quantify Off-Target Transgene Integration after CRISPR-Induced DNA Modification
Source: Methods Protoc. 2022 May 25;5(3):43. doi: 10.3390/mps5030043 (PMC9229199; doi:10.3390/mps5030043)
Supplement: Supplementary file 1 [file mps-05-00043-s001.zip › mps-1652437-supplementary.pdf]

Article

Validation of a Novel Double Control Quantitative Copy Number PCR Method to Quantify Off-Target Transgene Integration after CRISPR-Induced DNA Modification

Brit-Maren Michaud Schjeide [1,2]*, Maren Schenke [3], Bettina Seeger [3], and Gerhard Paul Püschel [1]

[1] Institute of Nutritional Science, Department of Nutritional Biochemistry, University of Potsdam, 14558 Nuthetal, Germany; gpuesche@uni-potsdam.de
[2] Department of Nutritional Biochemistry, Faculty of Life Sciences: Food, Nutrition and Health, University of Bayreuth, D-95326 Kulmbach, Germany; brit-maren.schjeide@uni-bayreuth.de
[3] Institute for Food Quality and Safety, Research Group Food Toxicology and Alternative/Complementary Methods to Animal Experiments, University of Veterinary Medicine Hannover, 30173 Hannover, Germany; Maren.Schenke@tiho-hannover.de; bettina.seeger@tiho-hannover.de
* Correspondence: brit-maren.schjeide@uni-bayreuth.de

Supplementary Materials: The following supporting information can be downloaded at: www.mdpi.com/xxx/s1, Table S1: Primers used for insert amplification, qPCR, dc-qcnPCR, and Southern blot; Figure S1: Southern blot with GLuc probe at 48 °C; Figure S2: Correlation between expression level rank and log of transgene copy number.

All primers are provided, which were used for the insert confirmation PCR, the gene expression analysis, the double control quantitative PCR, and the example Southern blot. The expected product size and annealing temperature are included (Supplementary Table 1).

Table S1: Primers used for insert amplification, qPCR, dc-qcnPCR, and Southern blot

Primer name	Sequence	Product size (bp)	Annealing temperature (°C)
**Gaussia luciferase**			
**GLuc_Fprobe3**	CCCCTTGATCTTGTCCACCT	299	60
**GLuc_Rprobe4**	GCACGCCCAAGATGAAGAAG		
**Autosomal control gene**			
**hCHOP-F**	CAGAACCAGCAGAGGTCACA	210	60
**hCHOP-R**	AGCTGTGCCACTTTCCTTTC		
**Chromosome X genes**			
**xRBBP7_F**	AAATTTCACTGACAGGGCCG	264	60
**xRBBP7_R**	GGCCATCTCAATTTGTCCCG		
**xGATA1_F**	CTGTTCTGGTAGCCTGTGGA	243	60
**xGATA1_R**	ACAGTTGAGGCAGGGTAGA		
**xHPRT1_F**	GGGCTAGACTTTTGAGGGACA	250	60
**xHPRT1_R**	AGTCCTAATCGGCCATTACTGA		
**xTMSB15B_F**	GTTGCTTTCAGTCTCTGCCC	244	60
**xTMSB15B_R**	GGGTAGCAGCAAACTCACAG		
**Homology directed repair genes**			
**hBRCA1_F**	CCAGGAGTGGAAAGGTCATCC	224	60
**hBRCA1_R**	TCCAGGTAAGGGGTTCCCTC		
**hRAD51_F**	CTGGGGCAAGCGAGTAGAGA	238	60
**hRAD51_R**	GCATTTATGCCACACTGCTCT		
**hMCPH1_F**	TGCTCTGGGTGGAAAAATGC	205	60
**hMCPH1_R**	TGCCTTTGTAGCTCTTTAGCCA		
**hMRE11_F**	TTGTATGGGTGATCGGCCTG	171	60
**hMRE11_R**	CAAAGTGCATCTGCCCCTGT		
**hDNA2_F**	GTCCCAATTGTGATGCTGCC	177	60
**hDNA2_R**	GCCACTCTTCTCCATTTCCGA		
**hEXO1_F**	AAGTGACCAGACCTCCAAGC	87	60
**hEXO1_R**	GACTTATATAGCCCAGGAACCTTGT		
**Reference genes**			
**PPIA-F**	GCCAAGACTGAGTGGTTGGAT	70	60
**PPIA-R**	GGCCTCCACAATATTCATGCC		
**RPS23-F**	ACAGGATGGGCAAGTGTCGT	75	60
**RPS23-R**	CACTTCTGGTCTCGTCGGTG		
**Insert confirmation PCR**			
**5'probeWT_F**	TTCAGGTTCCGTCTTCCTCC	850/3000	70
**Rpcr-wt-3'HA**	AGGATCCTCTCTGGCTCCAT		
**Southern blot example probe**			
**GLuc_F2**	GTCAGAACACTGCACGTTGG	395	60
**GLuc_R2**	AAGACTTCAACATCGTGGCC		

An example Southern blot with non-transfected SiMa gDNA as a negative control, SiMa Random-Insertion prototype clone gDNA as a positive control, as well as with two different SiMa CRISPR-modified clone gDNA digests (Supplementary Figure 1). gDNA

digests were labelled as non-transfected DNA (WT), SiMa Random-Insertion prototype DNA (1), SiMa CRISPR-modified clone 2 (2), SiMa CRISPR-modified clone 3 (3), and plasmid (Pl). Restriction enzymes used to digest the gDNA were: BglI (A), EcoRI (B), HindIII (C). White arrows indicate the expected sizes of the digested hPOMC-GLuc fragments. Southern blot was performed according to the DIG Application Manual for Filter Hybridization supplied by Roche. PCR preparation of southern blot probes was carried out using the Roche DIG PCR kit (sequences found in Supplementary Table 1) The example membrane was hybridized with the GLucF2R2 probe (395 bp) at 48 °C. As expected, no band was detected in the WT lane. Contrary to expectations, no band was detected in the SiMa prototype digests, despite the functionally proven stable integration of GLuc in the genome. Very faint bands were seen in the SiMa CRISPR-modified clone 2 digestion (2), all three different restriction enzyme digestions were at the expected size, but there was no indication of any off-target integrations. The second SiMa CRISPR-modified clone digestions (clone 3, lanes labelled 3) had extremely strong bands at the expected sizes as well as multiple unexpected bands at multiple sizes for each digest. The cut plasmid was included in this gel, which was stained with a strong band at 6.6 kbp, as expected (Supplementary Figure 1).

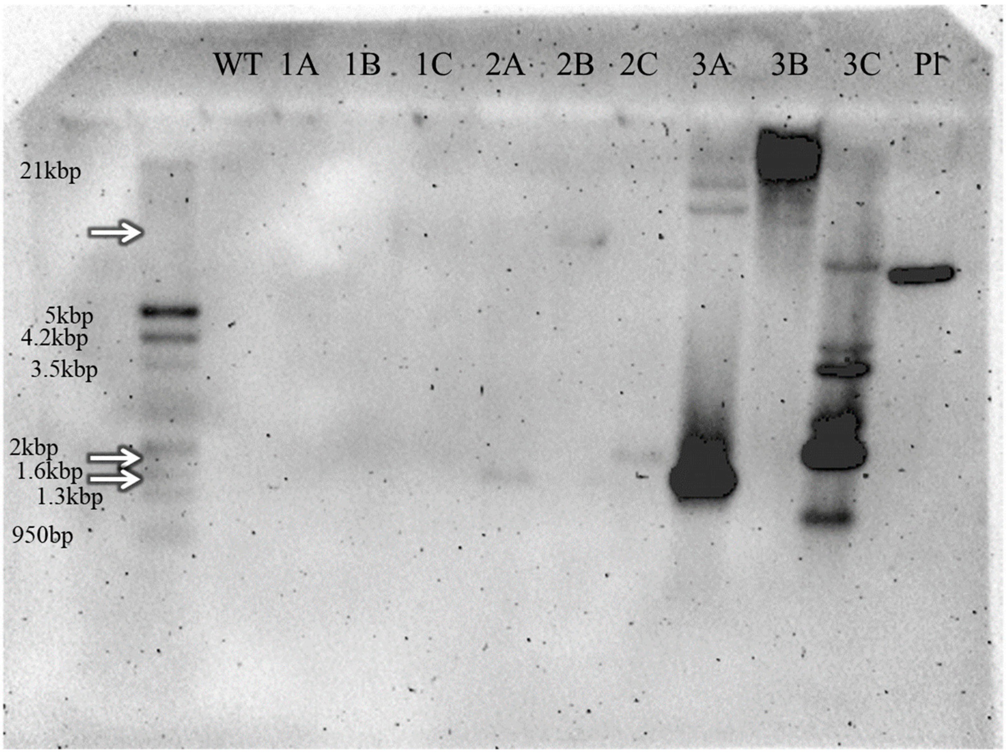

Figure S11: Southern blot with GLuc probe at 48 °C. WT = non-transfected SiMa gDNA, 1 = SiMa Random-Insertion prototype gDNA, 2 = SiMa CRISPR-modified clone 2 gDNA, 3 = SIMA CRISPR-modified clone 3 gDNA, A = BglI digestion (1.5 kbp), B = EcoRI digestion (10.3 kbp), C = HindIII digestion (1.8 kbp). Expected product sizes indicated with white arrows.

The functional correlation between double-strand break repair efficiency and the number of off-target transgene insertions was analyzed. The relative gene expression level of each of the repair enzymes was ranked and summed for each clone. There was a minor, but significant, negative correlation between the ranked expression levels of HDR-associated genes and the log of the transgene copy number of the analyzed clones (Pearson's correlation coefficient $r = -0.547$; $p < 0.05$) (Supplementary Figure 2).

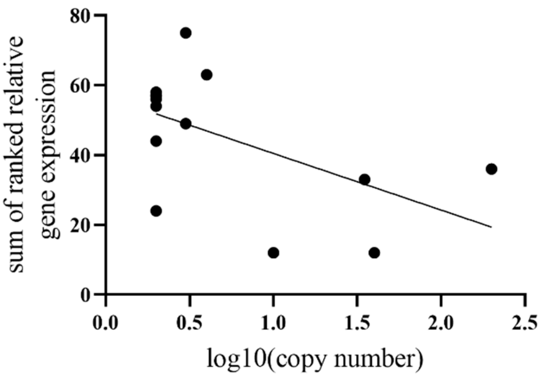

Figure S2: Functional correlation between ranked relative gene expression and transgene insert copy number. Statistical analysis: Pearson correlation coefficient, 95% confidence interval, two-tailed test. Pearson's correlation coefficient r = -0.547; $r^2 = 0.2992$; p = 0.043.
